# Viability and Adhesion of Periodontal Ligament Fibroblasts on a Hydroxyapatite Scaffold Combined with Collagen, Polylactic Acid–Polyglycolic Acid Copolymer and Platelet-Rich Fibrin: A Preclinical Pilot Study

**DOI:** 10.3390/dj10090167

**Published:** 2022-09-06

**Authors:** Leonor C. Espitia-Quiroz, Andrés L. Fernández-Orjuela, Lina M. Anaya-Sampayo, Adriana P. Acosta-Gómez, Luis Gonzalo Sequeda-Castañeda, Sandra Janeth Gutiérrez-Prieto, Nelly S. Roa-Molina, Dabeiba A. García-Robayo

**Affiliations:** 1 Resident in Periodontics, Dentistry Faculty, Pontificia Universidad Javeriana, Bogota 110231, Colombia; 2 Dentistry Faculty, Universidad Popular Autónoma del Estado de Puebla, Puebla 72410, Mexico; 3 Dentistry Research Center, Dentistry Faculty, Pontificia Universidad Javeriana, Bogota 110231, Colombia; 4 Periodontal System Department, Dentistry Faculty, Pontificia Universidad Javeriana, Bogota 110231, Colombia; 5 Chemistry Department, Sciences Faculty, Pontificia Universidad Javeriana, Bogota 110231, Colombia; 6 Dental System Department, Dentistry Faculty, Pontificia Universidad Javeriana, Bogota 110231, Colombia; 7 Oral System Department, Dentistry Faculty, Pontificia Universidad Javeriana, Bogota 110231, Colombia

**Keywords:** collagen, platelet-rich fibrin, fibroblasts, hydroxyapatite, periodontal ligament, tissue engineering, polylactic acid–polyglycolic acid copolymer

## Abstract

Background: Conventional periodontal therapy relies on bone regeneration strategies utilizing scaffolds made of diverse materials, among which collagen, to promote cell adhesion and growth. Objective: To evaluate periodontal ligament fibroblast (HPdLF) cell adhesion and viability for periodontal regeneration purposes on hydroxyapatite scaffolds containing collagen (HAp-egg shell) combined with polylactic acid–polyglycolic acid copolymer (PLGA) and Platelet-Rich Fibrin (PRF). Methods: Four variations of the HAp-egg shell were used to seed HPdLF for 24 h and evaluate cell viability through a live/dead assay: (1) (HAp-egg shell/PLGA), (2) (HAp-egg shell/PLGA + collagen), (3) (HAp-egg shell/PLGA + PRF) and (4) (HAp-egg shell/PLGA + PRF + collagen). Cell adhesion and viability were determined using confocal microscopy and quantified using central tendency and dispersion measurements; significant differences were determined using ANOVA (*p* < 0.05). Results: Group 1 presented low cell viability and adhesion (3.70–10.17%); groups 2 and 3 presented high cell viability and low cell adhesion (group 2, 59.2–11.1%, group 3, 58–4.6%); group 4 presented the highest cell viability (82.8%) and moderate cell adhesion (45%) (*p* = 0.474). Conclusions: The effect of collagen on the HAp-egg shell/PLGA scaffold combined with PRF favored HPdLF cell adhesion and viability and could clinically have a positive effect on bone defect resolution and the regeneration of periodontal ligament tissue.

## 1. Introduction

Osseous defects affect oral health in addition to being associated with unpleasant aesthetic aspects, favoring the progression of periodontal disease [1]. Recently, regenerative periodontal therapy has arisen as a new therapeutic option, whose objective is to grow new tissues on a previously diseased root surface to restore the damaged tissue, thus re-establishing the periodontal tissue functionality [2]. This can be achieved after the surgical implantation of biomaterials that, acting as scaffolds for epithelial cells, create a 3D environment with the biological characteristics that provide cells with an ideal milieu for proliferation and new tissue formation [3].

Tissue engineering is a new field aimed at the regeneration of tissues and organs by using biocompatible materials [3]. This principle is based on three pillars: cells, biomolecules and scaffolds. For scaffold construction, a wide range of materials exist, such as metals, ceramics and polymers. Scaffolds can be of natural or synthetic origin and reabsorbable or non-resorbable based on the nature of their components [4]. Among scaffolds, the most used are ceramics, such as dicalcium phosphate, β-tricalcium phosphate (β-TCP) and hydroxyapatite (HAp). The latter has been used in scaffold construction because of its osteoconductive properties, composition and similarities to bone [5,6]. However, HAp physical stability is questionable, hence diminishing the possibility of applying mechanical loads on it. Therefore, it is necessary to reinforce it with different materials that improve its flexibility and biodegradability [6]. One of the most used polymers is polylactic acid–polyglycolic acid copolymer (PLGA), which possesses high degradation rates, good mechanical properties, hardness and remarkable processability [7].

In addition to scaffolds, biomolecules are another pillar of tissue engineering. They include cellular adhesion peptides and growth factors that favor cell proliferation [3]. In addition, hemoderivatives, such as plasma rich in platelets (PRP), Platelet-Rich Fibrin (PRF) and plasma rich in growth factors are used to enhance tissue regeneration [5]. PRF is an autologous biomaterial, rich in active growth factors that can stimulate scar formation in wounds and bone formation [8,9]. Kapoor et al. [10] evaluated PRF on implant osteointegration, demonstrating PRF had a significant effect during the early stages of scar formation before introducing the implant. Other studies reported the successful use of this biomaterial to regenerate tissues [5,11,12,13,14] and its combination with polyvinyl alcohol and sodium alginate, acting as a scaffold and increasing cell proliferation and mineralization [15].

To date, our research group has carried out various studies in search of the ideal scaffold, evaluating grain size, purity, and crystallinity of synthetic and bovine HAps and of HAp obtained from the egg shell (HAp-egg shell), which share similar structures. Nevertheless, HAp-egg shell demonstrated additional advantages, such as being ecologically sustainable and readily available and having a low cost, properties which make it an ideal option [16,17,18]. HAp-egg shell/PLGA scaffolds have been also elaborated, reinforced with silicon, and osteoblastic cell viability on them was evaluated. The scaffolds obtained were highly porous and biocompatible, since they preserved cell morphology and 96% cell viability and adherence. Despite the advantages provided by silicon in relation to maintaining cell viability, it presented an increased solubility [17]. As a continuation of those studies, in an effort to pursue periodontal regeneration, human periodontal ligament fibroblasts (HPdLF) and osteoblasts were evaluated for cell viability and adhesion on HAp-egg shell/PLGA scaffolds combining them with PRF. The results demonstrated that cell viability and adhesion increased on this scaffold; however, the scaffold’s physical characteristics were not favorable [19].

In this last decade, collagen, an extracellular protein of natural origin, was included in scaffold construction [20,21,22] since it provides biocompatibility and aids in the combination of other materials. This last characteristic is indispensable in tissue regeneration, since it allows cell distribution and helps vessel growth in tissues [14,23,24,25].

Recent studies reported that collagen scaffolds combined with HAp improved bone formation, because they were readily absorbed. Likewise, studies demonstrated that collagen scaffolds, despite having mechanical properties similar to those of other scaffolds, when combined with HAp and other minerals improved cell proliferation by means of osteogenic and osteoimmunomodulation processes [26,27,28,29]. Hence, the aim of the present study was to investigate the possible beneficial effects of HAp-egg shell collagen scaffolds enriched with PLGA/PRF for PdLF cell adhesion and viability. The null hypothesis of our study was that there would be no difference in PDF cell adhesion if a PLGA/PRF collagen scaffold is utilized compared to a scaffold made only of HAp-egg shell and collagen. In the present study, a scaffold containing HAp-egg shell/PLGA/PRF/Col was developed to exploit the advantages offered by each single material, improve HPdLF cell viability and adhesion and provide possible new periodontal regeneration options.

## 2. Materials and Methods

### 2.1. Cell Culture

**Figure 1:**
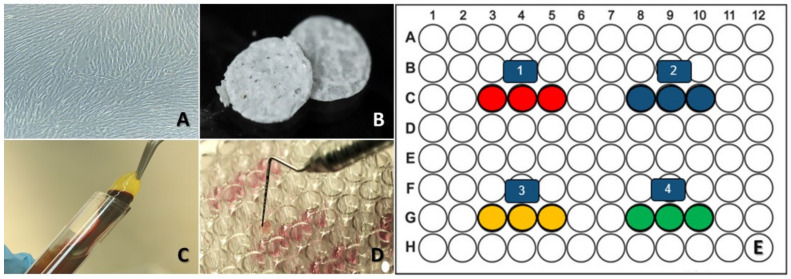
Experimental processes. (**A**) Human periodontal ligament fibroblasts culture; the cells show their characteristic spindle shape. (**B**) HAp-egg shell/PLGA scaffold used for all groups in the form of a 5 mm disc with 2 mm height. (**C**) Platelet-Rich Fibrin (PRF), with gelatinous consistency, a characteristic of this biomaterial. (**D**) Experimental design in a 96-well plate, containing the HPdLF culture medium. (**E**) Figure illustrating the four evaluated groups: red (HAp-egg shell/PLGA + HPdLF), blue (HAp-egg shell/PLGA + HPdLF + Collagen), yellow (HAp-egg shell/PLGA + HPdLF + PRF) and green (HAp-egg shell/PLGA + HPdLF + Collagen + PRF).

For cell culture, human periodontal ligament fibroblasts (HPdLF) (Clonetics™ Human Fibroblast Cell Systems) were cultured in a growth medium employed for stromal cells (SCGMTM Stromal Cell BulletKit™), purchased from Lonza^®^. In total, 350,000 cells/cm^2^ were seeded into T25cell culture flasks containing 5 mL of medium. The flasks were incubated at 37 °C and 5% de CO_2_, and the following day, the medium was changed; from then onwards, the medium was changed every two days (Figure 1A). The cells were subcultured when they reached 70–80% confluency.

### 2.2. HAp-Egg Shell/PLGA Scaffold Contribution

The scaffolds were elaborated using HAp synthesized from egg shell and modified with PLGA (HAp-egg shell/PLGA), utilizing a protocol previously standardized by our group (Figure 1B) [17].

### 2.3. Platelet-Rich Fibrin (PRF)

A blood sample from a 26-year-old male was collected after obtaining his signed consent, approved by the Pontificia Universidad Javeriana research ethics committee (Registry OD-0210. Act 006 from 21 April 2017). The donor was a healthy non-smoker who had not consumed any medication in the past three months. PRF was obtained following Chouckroun’s protocol [8,13]. Briefly, 6 tubes of blood without anticoagulant were collected and centrifuged at 3000 rpm for 10 min or 2700 rpm for 12 min. After centrifugation, the fibrinogen was concentrated in the middle part of the tube, where thrombin transformed it into fibrin, creating a clot. Red blood cells (RBCs) were located in the bottom of the tube, and an acellular plasma in the upper part. We collected the fibrin clot and the platelets, after removing them from the layer rich in RBCs (Figure 1C) [13,30].

### 2.4. Collagen

Collagen was purchased from Sigma -Aldrich (Collagen, Type I solution from rat tail) and prepared following the manufacturer’s instructions until attaining a 0.01% work concentration. The excess liquid was removed from the surface, and the sample left to dry overnight, followed washing it with a 0.9% saline solution and adding it to the growth medium and the cells [31].

### 2.5. Experimental Design

Four experimental groups were established using HAp scaffold discs obtained from egg shell modified with PLGA (common denominator in all groups); variations were based on the addition of collagen and/or PRF [30,31]. Collagen was added by submerging a disc into the collagen solution and allowing it to air-dry at room temperature (RT). PRF in the form of a clot was cut (Scalpel blade No. 15), obtaining a 2 mm fibrin mesh, which was added to the culture medium to embed the disc. Then, HPdLF were seeded on the discs to analyze their viability and adhesion [32]. The four established groups were: Group 1: HPdLF on HAp obtained from egg shell modified with PLGA (HAp-egg shell/PLGA + HPdLF); Group 2: HPdLF on HAp obtained from egg shell modified with PLGA and collagen (HAp-egg shell/PLGA + HPdLF + Collagen); Group 3: HPdLF on HAp obtained from egg shell modified with PLGA and PRF (HAp-egg shell/PLGA + HPdLF + PRF); Group 4: HPdLF on HAp obtained from egg shell modified with PLGA, PRF and collagen (HAp-egg shell/PLGA + HPdLF + Collagen + PRF). We made three replicates for each group, for a total of 12 assays in a 96-well plate (Figure 1D). Once all set ups were established, the cells were incubated for 24 h at RT.

### 2.6. Viability and Cell Adhesion Analysis

Cell viability and adhesion were determined through confocal fluorescent microscopy (CFM) with an Olympus FV1000 motorized inverted IX71 microscope using the LIVE/DEAD^®^ kit which contains calcein, an indicator of live cells through green fluorescence, and ethidium homodimer (EthD-1) an indicator of dead cells through red fluorescence. To this end, 20 µL of 2 mM EthD-1 was added to 10 mL of sterile PBS and mixed by vortex to obtain a 4 µM EthD-1 solution. Then, 5 µL of a 4 mM calcein solution dissolved in DMSO was added to the 4 µM EthD-1 solution and mixed by vortex. Subsequently, 100 µL of HPdLF cells was extracted from each well (Groups 1 to 4) and placed in separate tubes, and 150 µL of the LIVE/DEAD^®^ working solution was added, for a total of 250 µL. The tubes were incubated for 45 min at RT, and a sample from each tube was collected and placed on a microscope slide for observation under a confocal microscope.

### 2.7. Results Analysis

The confocal fluorescent microscope images were visualized and standardized with regard to contrast, brightness and saturation, using the Fluoview Ver.4.2b software (Olympus, New York, NY, USA). A 6 × 6 grid was superimposed on each image to count the cells in each grid, which consisted of a 5200 µm^2^ area. To determine cell adhesion, the total area of the image was divided by the total area of the grids with live and dead cells (green and red fluorescence), according to the following formula:

(1) $$Cell\;adhesion=\frac{5200\;µm^{2}\left(Living\;and\;dead\;cells\;areas\right)}{187,200\;µm^{2}}\times 100$$

To determine cell viability, the number of cells presenting green fluorescence was determined (viable cells) assuming 100% cell adhesion on the area evaluated. Therefore, cell viability was the percentage of green cells counted in comparison to the number of adherent cells. Any cell presenting red fluorescence representing the entire cell of part of it was considered dead.

A high, medium, low and null fluorescence scale was determined according to the fluorescence distribution in each grid (Table 1).

### 2.8. Data Statistical Analysis

All data were recorded in an Excel file and were analyzed using Stata v14 and GraphPad Prism v6 software. A descriptive analysis of the results was carried out on cell adhesion and viability using central tendency ($\bar{x}$: average) and dispersion (±: standard deviation) for each treatment evaluated [G1: (HAp-egg shell/PLGA + HPdLF); G2: (HAp-egg shell/PLGA + HPdLF + Collagen); G3: (HAp-egg shell/PLGA + HPdLF + PRF); G4: (HAp-egg shell/PLGA + HPdLF + Collagen + PRF] [32].

To verify the data’s normal distribution, a Shapiro–Wilk test was performed. Then, a comparison of each variable with respect to each treatment group (G1, G2, G3 and G4) was performed using one-way ANOVA with a Bonferroni post-hoc test. Significance was established at *p* < 0.05 [32].

## 3. Results

**Figure 2:**
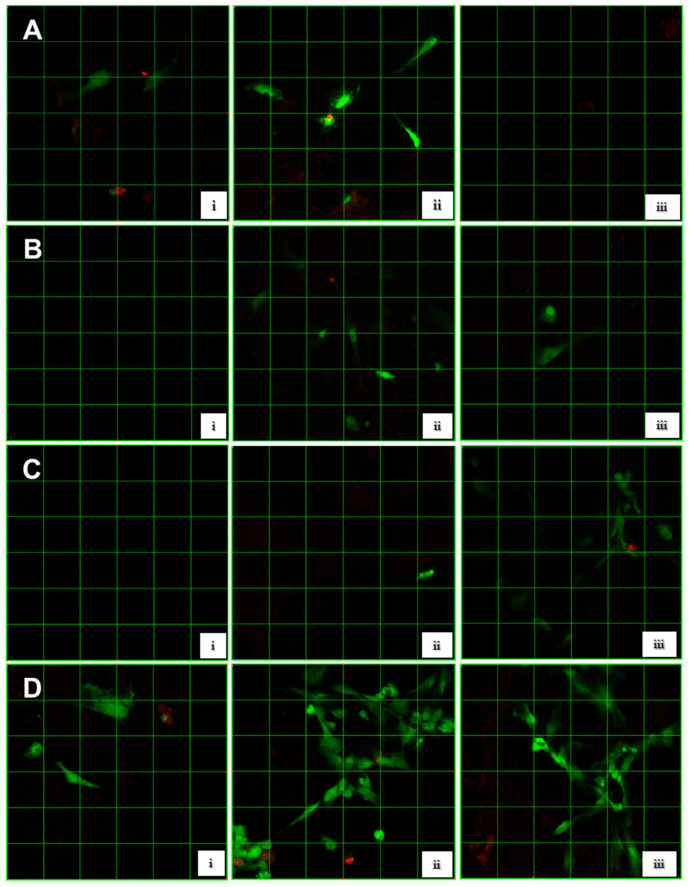
Images captured under a confocal fluorescence microscope (CFM); *n* = 3 (i, ii, iii). (**A**). HAp-egg shell/PLGA + HPdLF. (**B**). HAp-egg shell/PLGA + HPdLF + Collagen. (**C**). HAp-egg shell/PLGA + HPdLF + PRF. (**D**). HAp-egg shell/PLGA + HPdLF + PRF + Collagen. Live cells are green, dead cells are red.

Three images were obtained from CFM analysis, for a total of 12 images (Figure 2).

Group 1 (Figure 2A): HPdLF seeded onto the HAp-egg shell disc modified with PLGA.

In Figure 2A(i), no cells were observed; thus, no cell adhesion and viability was identified. In Figure 2A(ii), spindle-shaped fibroblasts were observed containing both green and red fluorescence and displaying 75% viability and 11.1% adhesion. Figure 2A(iii) shows only red fluorescence, indicating no viable cells (0%), with low cell adhesion (19.4%). On average, group 1 showed cell viability and adhesion of 25% and 10.17%, respectively (Table 2).

Group 2 (Figure 2B): HPdLF seeded onto the HAp-egg shell disc containing PLGA and collagen.

No cells are observed in Figure 2B(i); therefore, no cell adhesion and viability was identified. In Figure 2B(ii), a round fibroblast was observed with red fluorescence, as well as green-fluorescent spindle-shaped fibroblasts, indicating 77.7% viability and 25% adhesion. In Figure 2B(iii), a very high viability was obtained (100%), as indicated by the green-fluorescent spindle-shaped fibroblast, with low adhesion (8.3%). On average, group 2 showed viability and cell adhesion of 59.2% and 11.1%, respectively (Table 2).

Group 3 (Figure 2C): HPdLF seeded onto the HAp-egg shell disc containing PLGA and PRF.

In Figure 2C(i), no cells were observed; hence, no cell viability or adhesion was observed. In Figure 2C(ii), a green-fluorescent round fibroblast was observed, indicating 100% viability and 2.7% adhesion. In Figure 2C(iii), 75% cell viability was measured, with 11.1% adhesion. On average, group 3 showed 58% viability and 4.6% cell adhesion (Table 2).

Group 4 (Figure 2D): HPdLF seeded onto the HAp-egg shell disc containing PLGA, PRF and collagen.

In Figure 2D(i), 88.8% viability was measured, with 25% cell adhesion. In Figure 2D(ii), 77.7% viability and 50% adhesion were observed. In Figure 2D(iii), 81.8% viability and 61.1% were measured. All images showed spindle-shaped fibroblasts with predominant green fluorescence. On average, group 4 showed 82.8% viability and 45% cell adhesion (Table 2).

One-way ANOVA with Bonferroni post-hoc test revealed that the cell adhesion percentage was significantly different in group 4 with respect to the other groups (*p* = 0.015). In contrast, no statistically significant differences were observed for cell viability among the evaluated groups (*p* = 0.474).

**Figure 3:**
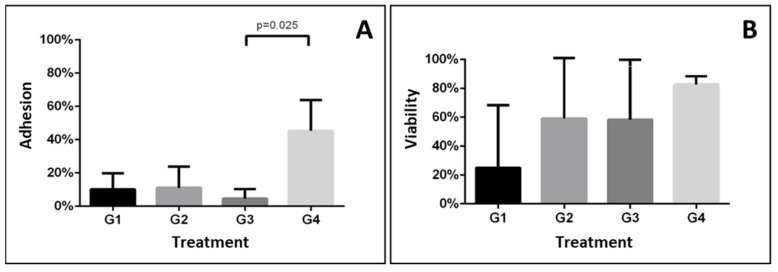
Comparison among the evaluated groups of cell adhesion (**A**) and viability (**B**). Mean ± SD.

In fact, post-hoc comparisons among all groups revealed significant differences between groups 3 and 4 (*p* = 0.025). On the other hand, cell viability was similar among all groups evaluated (Figure 3).

## 4. Discussion

Biomaterials have been widely used in periodontal regeneration as well as in the restoration of compromised dentitions with dental implants [33,34]. Likewise, biomaterials have been used in deciduous teeth restoration affected by cavities, where in the long term, an HAp interphase between the dental tissue and the biomaterial should be observed, characteristic of these biomaterials [35]. However, biomaterials are mostly used in tissue regeneration [36]. Periodontal regenerative therapy consists in osseous defect repair utilizing components based on tissue engineering. The elements used in this technique are principally biomaterials that allow the construction of scaffolds, which must be suitable to sustain cell viability, key in tissue regeneration. In this study, HPdLF cell adhesion and viability were evaluated in vitro after the cells were seeded onto an HAp-egg shell/PLGA and PRF scaffold containing collagen as a biomaterial.

In vitro and in vivo studies reported the use of scaffolds elaborated with materials that contained calcium phosphate and showed their contribution to the adhesion, differentiation and proliferation of osteoblasts and mesenchymal stem cells [17,18,37]. HAp obtained from egg shell has proven to provide good results in bone tissue regeneration, due to its characteristics, such as biocompatibility, bioactivity and osteoconductive properties [17,38]. In the present study, by means of the Live/Dead^TM^ assay, it was possible to observe simultaneously a green stain with calcein AM, indicating intracellular esterase activity, and a red stain with ethidium homodimer, indicating the loss of plasma membrane integrity. Hence, cells with green fluorescence were considered viable, whereas cell death was indicated by the presence of red fluorescence. Additionally, cells presenting both green and red fluorescence were also non-viable cells, because of plasma membrane integrity loss [39]. The images of to Group 1 obtained by CFM (HAp-egg shell/PLGA + HPdLF) (Figure 2A) demonstrated poor cell viability (25%) and low cell adhesion (10.7%, Table 2). These results are in agreement with our previous study [19], where diminished cell viability was observed in the absence of PRF. However, in a study performed by Gutiérrez et al. [17], 96% osteoblastic viability was obtained on an HAp-egg shell/PLGA scaffold reinforced with silicon. The authors suggested that silicon on the scaffold had a significant effect on cell viability, since it could potentiate HAp properties; yet, it should be noted that PLGA could trigger an inflammatory response in patients and silicon could affect the HAp-egg shell/PLGA scaffold solubility. Therefore, it seems the HAp-egg shell/PLGA scaffold by itself does not provide the favorable biological conditions for cell proliferation required in a tissue regeneration process.

A criterion to take into account in scaffold fabrication for tissue regeneration purposes is cell viability evaluation in a 3D culture. To this end, diverse techniques exist, and staining with ethidium homodimer and calcein AM (EthH/CaAM), known as the Live/Dead^TM^ assay is the most widely used. This technique allows the evaluation directly on the scaffold of cell morphology and distribution; furthermore, under confocal microscopy, it can distinguish between viable cells (green cells) and dead cell (red cells) detected by confocal microscopy. However, as disadvantages, it should be pointed out that the microscope must be calibrated to eliminate the possible scaffold’s autofluorescence. Additionally, cell proliferation cannot be analyzed, as other markers would be required, such as Ki-67 [40,41]. Other authors mention the use of techniques, such as metabolic reduction of resazurin into resorufin. Even though these are low-cost assays, easy to perform, they are not precise for 3D cultures, as they require the construction of standard curves to calculate cell viability. If the number of cells is high, then the metabolic reduction is slow, resulting in an underestimation of live cells. Moreover, these techniques do not allow the visualization of cell morphology. Last, it must also be considered that techniques which evaluate cell metabolism could vary in efficiency depending on the cell type, such as when evaluating stem cell differentiation on scaffolds [42].

In the CFM images of group 2 (HAp-egg shell/PLGA + HPdLF + Collagen) (Figure 2B), 59.2% viability and 11.1% cell adhesion were obtained (Table 2). In comparison to group 1 (HAp-Egg Shell/PLGA + HPdLF), an increase in cell viability was observed; however, cell adhesion did not improve. The literature reports that collagen provides the necessary elasticity for cells to migrate and produce extracellular matrix, including collagen fibers [43,44]. Our results are in agreement with those obtained by Jin et al. [22], who seeded bone marrow stem cells (BMSCs) and human gingival fibroblasts (HGF) onto a PLGA scaffold reinforced with fish collagen (FC) and bioactive nanohydroxyapatite (nHAp) for guided bone regeneration. They observed that the viability of both cell types was higher on FC and nHAp scaffolds in comparison with PLGA scaffolds, since FC and nHAp facilitated cell adhesion and proliferation. In addition, they concluded that FC significantly improved the mechanical forces and accelerated the degradation of PLGA constructed membranes, i.e., they potentiated their biodegradability.

In this study, PRF was used due to its biological properties and because it can be obtained at a low cost. Moreover, obtaining PRF for research purposes is fast and easy and does not require the addition of other supplements or anticoagulants. In a clinical setting, PRF represents an autologous source of growth factors, such as PDGF, VEGF, IGF, TGF-B, gradually released over a 10-day period [45]. In the clinic, its use has been successful in promoting hard and soft tissue regeneration [8,46]. Likewise, in the treatment of controlled periodontal disease, the combination of PRF with autologous osseous tissue reduces the pocket probing depth. Moreover, it results in significant bone gain improvement at the site of osseous defect, compared with autologous bone alone [47].

Then, PRF was included in our study as a biological inducer, which provides nutrients to the scaffolds since it releases growth factors in a controlled and progressive manner. It is also known to potentiate cell metabolism in various connective tissues [48,49]. In the CFM images of group 3 (HAp-egg shell/PLGA + HPdLF + PRF) (Figure 2C), 58% viability was obtained, with low cell adhesion (4.62%); these values were similar to those of group 2 (HAp-egg shell/PLGA + HPdLF + Collagen) and to those obtained in previous studies [19,22], which confirmed that cell activity increased in the presence of PRF, despite the size and form of the fibrin mesh used in this study, which did not allow complete cell contact with the scaffold’s surface. Therefore, in future studies, this must be taken into account.

In the literature, few studies have evaluated the behavior of PRF in the presence of collagen. Ghanaati et al. [50] used a xenotransplant, PRF and collagen matrices in seven patients with alveolar ridge atrophy. After four months, they did not find scars or fibrotic zones in the soft tissue. Additionally, after eight months, they observed histologically new bone formation with adequate vascularization when the implants were introduced. Another study for maxillary sinus floor elevation in 12 patients used PRF injected into collagen sponges. On an X-ray imaging control performed six months after the procedure, significant bone formation at all sites was observed [51].

In the present study, the CFM images of group 4 (HAp-egg shell/PLGA + HPdLF + Collagen + PRF) (Figure 2D) revealed a great number of fibroblasts with predominant green fluorescence in comparison with the other groups, suggesting that the collagen + PRF combination had a synergistic effect favoring an optimal environment for cell survival. In Figure 2D(iii), we also observed that fibroblasts produced protrusions, creating a meshwork, which could suggest interaction among cells, and displayed a very high viability (82.8%), which could be promoted by a moderate cell adhesion (45%) and was statistically significant in comparison with the results obtained for all other experimental groups (Table 2 and Figure 3).

A study by Lin et al. [52] investigated the potential migration, cell adhesion and tissue repair of oral fibroblasts and periodontal ligament in the presence of four collagen matrices. Their results demonstrated the collagen matrices had a favorable influence on oral cell behavior. Additionally, it was reported that different collagen types regulate cell migration, including fibroblast migration, as they interact with various integrins on the cell surface [53]. Moreover, they are highly biocompatible, generating an adequate microenvironment for cell growth [54]. These studies are in agreement with the results herein obtained, suggesting that the collagen applied to the (HAp-eggshell/PLGA + HPdLF + Collagen + PRF) scaffold favored not only the proper scaffold’s structural features but also proper biological functions. A limitation of this study is the low number of biological replicas; therefore, for validation in clinical studies, this should be considered in future studies.

## 5. Conclusions

The results of this study suggest that collagen application to the HAp-egg shell/PLGA scaffold in the presence of PRF improved the scaffold’s structural characteristics, in such a way that it could potentiate cell adhesion and at the same time sustain HPdLF viability. Specifically, the improvement of HAp modifications with other materials and using advanced technologies, such as 3D impression, would greatly contribute to the application of innovative materials in a clinical setting.

## Figures and Tables

**Table 1:** Fluorescence scale on performed measurements.

Scale	Area	Percentage
Null	0 µm^2^	0%
Low	1 µm^2^–46,800 µm^2^	1–25%
Moderate	46,801µm^2^–93,600 µm^2^	26–50%
High	93,601 µm^2^–140,400 µm^2^	51–75%
Very high	140,401 µm^2^–187,200 µm^2^	76–100%

**Table 2:** Cell viability and adhesion measured for the four experimental groups (*n* = 3).

Group	Adhesion					Viability				
	1	2	3	Average	*p*-Value	1	2	3	Average	*p*-Value
1	0.0	11.1	19.4	10.2 ± 9.7	0.015	0.0	75.0	0.0	25.0 ± 43.3	0.474
2	0.0	25.0	8.3	11.1 ± 12.7		0.0	77.7	100.0	59.2 ± 52.5	
3	0.0	2.7	11.1	4.6 ± 5.8 ^a^		0.0	100.0	75.0	58.3 ± 52.0	
4	25.0	50.0	61.1	45.4 ± 18.5 ^b^		88.8	77.7	81.8	82.7 ± 5.6	

Group 1: HPdLF seeded onto an HAp-egg shell disc modified with PLGA. Group 2: HPdLF seeded onto an HAp-egg shell disc with PLGA and collagen. Group 3: HPdLF seeded onto an HAp-egg shell disc with PLGA and PRF. Group 4: HPdLF seeded onto an HAp-egg shell disc with PLGA and PRF and collagen; *n* = 3. One-way ANOVA with Bonferroni post-hoc test: ^a^ significant difference with respect to G4; ^b^ Significant difference with respect to G3.

## Data Availability

The datasets used and/or analyzed during the current study are available from the corresponding author on reasonable request.
